# Draft genome of *Prochlorothrix hollandica* CCAP 1490/1^T^ (CALU1027), the chlorophyll *a/b-*containing filamentous cyanobacterium

**DOI:** 10.1186/s40793-016-0204-4

**Published:** 2016-10-18

**Authors:** Natalia Velichko, Mikhail Rayko, Ekaterina Chernyaeva, Alla Lapidus, Alexander Pinevich

**Affiliations:** 1 Department of Microbiology, Faculty of Biology, St. Petersburg State University, St. Petersburg, Russia; 2 Theodosius Dobzhansky Center for Genome Bioinformatics, St. Petersburg State University, St. Petersburg, Russia; 3 Center for Algorithmic Biotechnology, St. Petersburg State University, St. Petersburg, Russia

**Keywords:** Cyanobacteria, Prochlorophytes, *Prochlorothrix hollandica*, Comparative genomics

## Abstract

*Prochlorothrix hollandica* is filamentous non-heterocystous cyanobacterium which possesses the chlorophyll *a*/*b* light-harvesting complexes. Despite the growing interest in unusual green-pigmented cyanobacteria (prochlorophytes) to date only a few sequenced genome from prochlorophytes genera have been reported. This study sequenced the genome of *Prochlorothrix hollandica* CCAP 1490/1^T^ (CALU1027). The produced draft genome assembly (5.5 Mb) contains 3737 protein-coding genes and 114 RNA genes.

## Introduction

The majority of cyanobacteria use chl *a* as a sole magnesium tetrapyrrole and common phycobilisome functioning as the bulk LHC. The prochlorophytes are a unique pigment subgroup of phylum *Cyanobacteria* – besides chl *a*, they contain other chls (*b*; 2,4-divinyl *a*; 2,4-divinyl *b*; *f*; *g*) as antennal pigments and simultaneously do not depend on the PBP-containing photoreceptors [1]. Prochlorophytes demonstrating these outgroup features are few and encompass three marine unicellular genera (*Prochloron*, *Prochlorococcus*, *Acaryochloris*) and one freshwater filamentous (*Prochlorothrix*). Unicellular *Prochlorococcus* spp. dominate in phytoplankton of oligotrophic regions of the world’s ocean and they are of exceptional importance from the viewpoint of global primary productivity [2]. *Prochloron* sp. and *Acaryochloris* sp. were isolated in symbiotic association with colonial ascidians [3, 4]. In contrast to other prochlorophytes distribution, *P. hollandica* is characterized by low abundance and patchy distribution [5]; more detailed genome analysis would explain the ecophysiological background of this microorganism.

The genus *Prochlorothrix* is represented by two cultivable free-living species: *Prochlorothrix hollandica* and *Prochlorothrix* *scandica*, as well as a number of unculturable strains, originating from environmental 16S rRNA sequences [6]. The distinction between *P. hollandica* and *P. scandica* is predominantly based on the molecular-genetic characters: DNA reassociation less than 30 % and DNA GC mol% content difference more than 5 % [5].

*P. hollandica* was isolated from the water bloom of Loosdrecht lake (near Amsterdam, Nertherlands) and validly published under the rules of Bacteriological Code as the type strain CCAP 1490/1^T^ [7, 8]. The strain CCAP 1490/1 was generously supplied in 1994 by Dr. Hans C.P. Matthijs (Amsterdam University) and since then stored as CALU1027 at the Collection of Cultures of Algae and Microorganisms of St. Petersburg State University, CALU [9]. *Prochlorothrix hollandica* is also maintained as different strains under collection indexes CCMP34, CCMP682, NIVA-5/89, SAG10.89, and the strain PCC9006 was reported as well [10]. Another filamentous strain *Prochlorothrix* *scandica* was isolated from the phytoplankton of Lake Mälaren (Sweden), and is maintained as NIVA-8/90 and CALU1205 [11].

Among prochlorophytes at first were sequenced small genomes of unicellular *Prochlorococcus* sp. strains from LL- and HL-clades [2, 12, 13]. Four sequenced genomes of symbiotic *Prochloron didemni* P1-P4 are second in number [14]. *Acaryochloris marina* genomes were sequenced in the strains CCME5410 and MBIC11017 [15], but only one paper mentioned about *P. hollandica* PCC9006 genome sequenced by Shich et al. in the context of improving of global cyanobacterial phylogeny [16]. Here we report that genomic DNA of *P. hollandica* CCAP 1490/1^T^ (CALU1027) was sequenced and obtained draft genome was annotated in order to conduct investigations in the field of comparative genomics of cyanobacteria and prochlorophytes.

## Organism information

### Classification and features

**Fig. 1:**
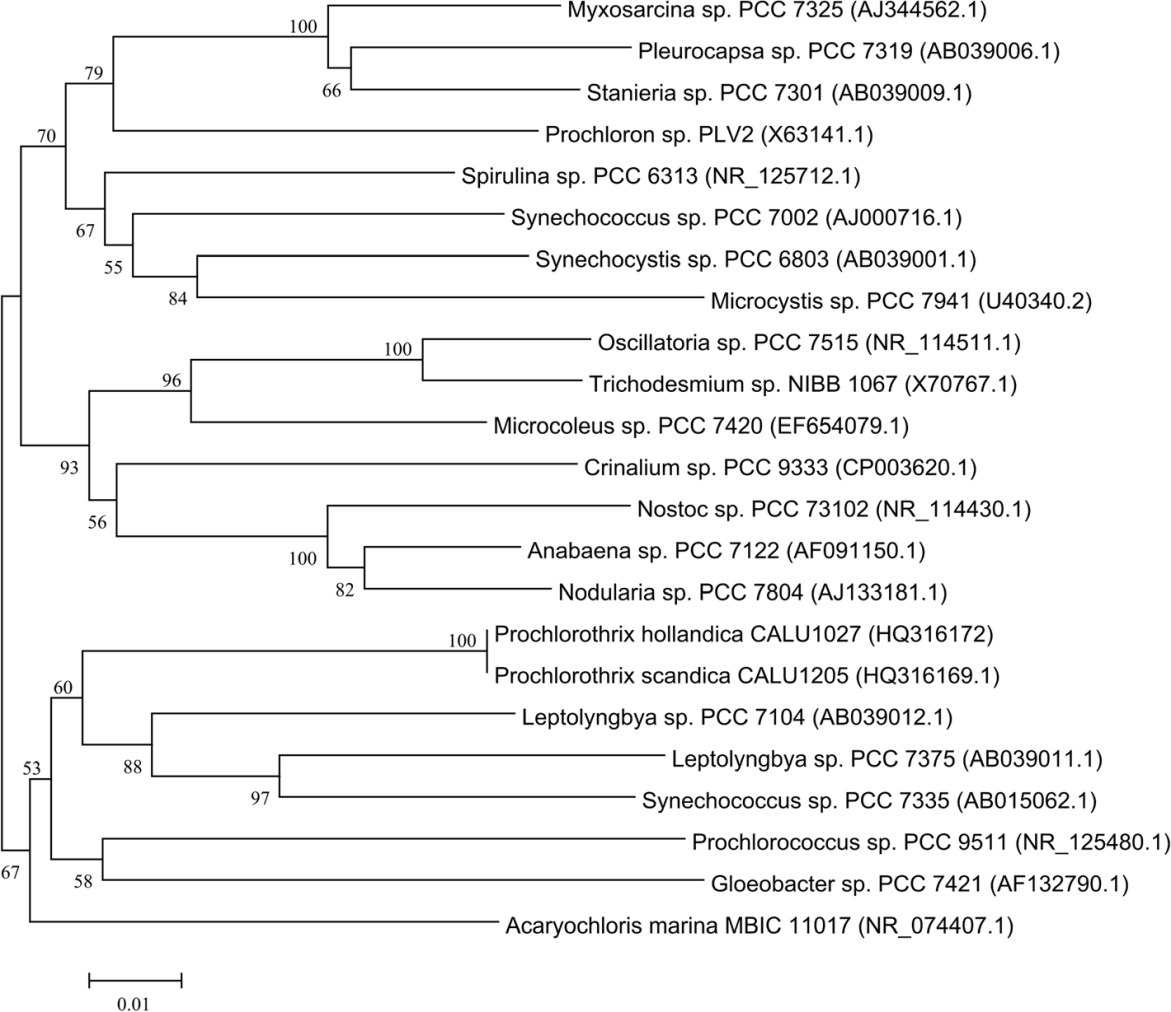
Phylogenetic position of *P. hollandica* CALU1027 within cyanobacteria. GenBank accession numbers are indicated in parentheses. The numbers above branches indicate bootstrap support from 1000 replicates

**Fig. 2:**
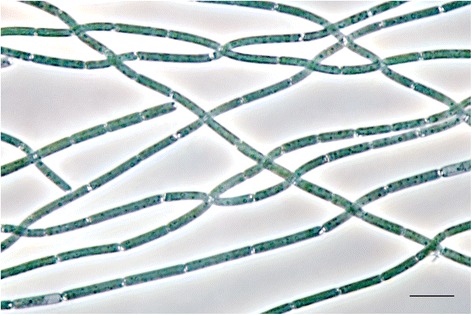
Light micrograph of *P. hollandica* CALU1027. Scale bar 10 μm

A representative genomic 16S rDNA sequence of strain *P. hollandica* CCAP 1490/1^T^ (CALU1027) was compared with another prochlorophytes and also with cyanobacterial type strains sequences obtained from GenBank. The tree was reconstructed using neighbor-joining with the Kimura-2 parameter substitution model in MEGA 6.0 [17, 18]. The phylogenetic position of *P. hollandica* CALU1027 represents in Fig. 1. Representatives of the genus *Prochlorothrix* are morphologically similar to other filamentous non-heterocystous cyanobacteria (Subsection III, *Oscillatoriales*) [19]. In particular, *P. hollandica* CALU1027 produces long (>300 μm) straight, unbranched, non-motile trichomes (Fig. 2). Individual cells are 1.6 ± 0.1 μm wide and 11.8 ± 0.9 μm long that matches with the data reported [2, 4]. The opaque polar aggregates of gas vesicles resemble of those presented in *Pseudanabaena* type, but *P. hollandica* trichomes possess more slight intercellular constrictions (1/5 − 1/8 cell diameter). Trichomes multiply by means of occasional breakage without the resulting formation of hormogonia. Light- or electron microscopic-visible sheath and mucilaginous capsule were never observed; cell envelope demonstrates a typical Gram-negative triple-layer contour [5]. A brief survey of *P. hollandica* CALU1027 properties according to MIGS recommendations [20] is given in Table 1.

**Table 1:** Classification and general features of *P. hollandica* CALU1027

MIGS ID	Property	Term	Evidence code^a^
	Current classification	Domain *Bacteria*	TAS [33]
		Phylum BX *Cyanobacteria*	TAS [19]
		Class *Photobacteria*	TAS [34]
		Order *Prochlorales*	TAS [34]
		Family *Prochlorothrichaceae*	TAS [8]
		Genus *Prochlorothrix*	TAS [8]
		Species *Prochlorothrix hollandica*	TAS [8]
		Type strain CCAP 1490/1^T^	TAS [8]
	Gram stain	Not reported	
	Cell shape	Elongated rods	TAS [5, 8]
	Motility	Nonmotile	TAS [8]
	Sporulation	Not reported	
	Temperature range	15 °C − 30 °C	TAS [8]
	Optimum temperature	20 °C	TAS [5, 8]
	pH range, Optimum	8.4	TAS [8]
	Carbon source	Autotroph	TAS [8]
	Energy source	Phototroph	TAS [8]
MIGS-6	Habitat	Freshwater	TAS [8]
MIGS-6.3	Salinity	Less than 25 mM	TAS [5, 8]
MIGS-22	Oxygen requirement	Aerobic	TAS [8]
MIGS-6.4	Chlorophyll type	Chlorophylls *a* and *b*	TAS [8]
MIGS-15	Biotic relationships	Free-living	TAS [8]
MIGS-14	Pathogenicity	Not reported	
MIGS-4	Geographic location	Loosdrecht lake, The Netherlands	TAS [8]
MIGS-5	Sample collection time	9 July, 1984	TAS [8]
MIGS-4.1	Latitude	52.20 N	TAS [8]
MIGS-4.2	Longitude	5.5 E	TAS [8]
MIGS-4.3	Depth	0.2 m	TAS [8]
MIGS-4.4	Altitude	2 m	NAS

^a^Evidence codes - *TAS* Traceable Author Statement (i.e., a direct report exists in the literature), *NAS* Non-traceable Author Statement (i.e., not directly observed for living, isolated sample, but based on a generally accepted property for the species, or anecdotal evidence)

These evidence codes are from the Gene Ontology Project [25]

## Genome sequencing information

### Genome project history

The WGS project AJTX02 has been deposited at DDBJ/EMBL/GenBank under accession AJTX00000000 (20.02.2013) and updated, in this research, as Draft Genome Project AJTX00000000.2 (29.04.2015). The assembled contigs have been deposited in NCBI. The project information and its association with the MIGS are summarized in Table 2.

**Table 2:** Project information

MIGS ID	Property	Term
MIGS-31	Finishing quality	Draft
MIGS-28	Libraries used	Illumina paired-end library
MIGS-29	Sequencing platform	Illumina MiSeq
MIGS-31.2	Fold coverage	30×
MIGS-30	Assemblers	SPAdes v. 3.5.0
MIGS-32	Gene calling method	GeneMarkS+
	Locus Tag	PROH
	GenBank ID	GCA_000341585.2
	Genbank date of release	20 February, 2013
	Gold ID	Gp0010359
	BioProject	PRJNA63021
	DDBJ ID	AJTX00000000.2
MIGS-13	Source Material Identifier	CALU1027
	Project relevance	comparative genomics

### Growth conditions and genomic DNA preparation

*P. hollandica* CALU1027 was grown in the BG-11 medium [2]. The strain is a moderate mesophile, well growing at 20-22 °C under continuous flux of light. For DNA isolation cells were harvested by centrifugation and treated with 2 μg/mL Proteinase K in 0.1 M Tris-HCl (pH 8.5), 1.5 M NaCl, 20 mM Na_2_EDTA, and 2 % cetyltrimethylammonium bromide at 55 °C for 3-4 h. DNA was purified by standard protocol of organic extraction and ethanol precipitation.

### Genome sequencing and assembly

For genome sequencing, DNA was randomly fragmented using Q800R sonicator system. After size selection, 500 bp DNA fragments were used for constructing sequence libraries and thereafter sequenced with a 250 bp paired-end reads method using the Illumina MiSeq platform according to the manufacturer’s protocol, resulting in 3,679,738 read pairs. Reads were processed via the Trimmomatic 0.32 tool [21] and after filtration there were 3,665,348 read pairs. The obtained reads were used for further genome assembly with SPAdes 3.5 [22]. From the resulting assembly, the *P. hollandica* CALU1027 contigs was selected and scaffolded with Contiguator 2.7.4 [23], using assembly GCF_000332315.1 as a reference. The draft genome of *P. hollandica* CALU1027 contained about 5.5 Mbp in 286 contigs organized in 10 scaffolds; the N50 length of the contigs was 33,173 and N50 length of the scaffolds - 1,244,169 bp (Table 3).

**Table 3:** Genome statistics

Attribute	Genome (total)	
	Value	% of total^a^
Genome size (bp)	5,525,469	100.00
DNA coding (bp)	3,931,877	71.16
DNA G + C (bp)	2,999,78	54.56
DNA scaffolds	10	−
Total genes	4,294	100.00
Protein coding genes	3,737	87.00
RNA genes	57	1.32
rRNA genes	12	0.28
tRNA genes	44	1.02
ncRNA genes	1	0.02
Pseudo genes	515	11.99
Genes in internal clusters	235	5.4
Genes with function prediction	2,770	64,5
Genes assigned to COGs	2,855	66.00
Genes with Pfam domains	2,386	55.56
Genes with signal peptides	86	2
Genes with transmembrane helices	869	20.24
CRISPR repeats	9	0.2

^a^ The total is based on either the size of the genome in base pairs or the total number of protein coding genes in the annotated genome

### Genome annotation

Protein-coding genes of draft genome assembly were predicted using the NCBI Prokaryotic Genome Annotation Pipeline (v.2.10) and an annotation method of best-placed reference protein set with GeneMarkS+ [24]. The annotated features were genes, CDS, rRNA, tRNA, ncRNA, and repeat regions. Functional assignments of the predicted ORFs were based on a BLASTP homology search against WGS of phylogenetically closest cyanobacteria and the NCBI non-redundant database. Functional assignment was also performed with a BLASTP homology search against the Clusters of Orthologous Groups (COG) database [25, 26]. As much as 2855 genes (66 %) were assigned as a putative function, and the remaining genes were annotated as either hypothetical proteins or proteins with unknown function.

## Genome properties

The GC content of the *P. hollandica* CALU1027 genome was 54.56 %. Gene annotation revealed 3737 protein coding genes, 12 rRNA genes, and 44 tRNA genes. COG annotations of protein coding genes are presented in Table 4.

**Table 4:** Number of genes associated with general COG functional categories

Code	Value	% age^a^	Description
J	160	4.28	Translation, ribosomal structure and biogenesis
A	0	0	RNA processing and modification
K	141	3.77	Transcription
L	213	5.69	Replication, recombination and repair
B	3	0.08	Chromatin structure and dynamics
D	39	1.04	Cell cycle control, cell division, chromosome partitioning
V	64	1.71	Defense mechanisms
T	316	8.46	Signal transduction mechanisms
M	210	5.62	Cell wall/membrane biogenesis
N	56	1.50	Cell motility
U	76	2.03	Intracellular trafficking and secretion
O	144	3.85	Posttranslational modification, protein turnover, chaperones
C	148	3.96	Energy production and conversion
G	126	3.37	Carbohydrate transport and metabolism
E	201	5.37	Amino acid transport and metabolism
F	67	1.79	Nucleotide transport and metabolism
H	156	4.17	Coenzyme transport and metabolism
I	55	1.47	Lipid transport and metabolism
P	136	3.64	Inorganic ion transport and metabolism
Q	52	1.39	Secondary metabolites biosynthesis, transport and catabolism
R	407	10.89	General function prediction only
S	409	10.94	Function unknown
−	8	0.21	Not in COGs

^a^The total is based on the total number of protein coding genes in annotated genome

## Insights from the genome sequence

The assembly and analysis of *P. hollandica* CALU1027 genome annotation revealed a repertoire of genes necessary for the autonomous energy and substrate metabolism: 743 detected genes with relevance to 129 metabolic pathways have orthologs in *P. hollandica* CALU1027 and other cyanobacteria (Table 5). Comparative genomes analysis of *P. hollandica* CALU1027 with filamentious heterocystous cyanobacteria *Anabaena variabilis* ATCC29413 and unicellular prochlorophytes *Prochlorococcus marinus* CCMP1375 and *Acaryochloris marina* MBIC11017 revealed that the main differences were in the amino acids compounds, carbohydrates metabolism, membrane transport and stress response systems (data not shown).

**Table 5:** Selected functional capacities

Cell function	Metabolic system/element	Putative gene/gene product
Light energy metabolism	Oxygenic phototrophy; photorespiration	*psaA-F*, *psaJ-L*, *psaX*, *psbA-D*, *psbH-P*, *psbU*, *psbV*, *psbW*, *psbZ, pcbA*-*C*; ycf39, *petA*, *petB*, *pet D*, *petE*, *hoxH*/*hoxY*, PsbF, cyt *f*, cyt *b*_6_; PC, CAT, SGAT, Fd-GOGAT, IpdA
Dark energy metabolism	Glycolysis and gluconeogenesis; methylglyoxal metabolism, pentose phosphate pathway; Entner-Doudoroff pathway; pyruvate cleavage; TCA with glyoxylate bypass	GlcK, HxK, PPgK, PfK1, PfK2, PPiFKa, PPiFKb, Fbp_I, B, X; Fba1- 2, TpI, GADH, G3PNP, PgK, PgM, EnO, PyK, PpS, PpD, Hyp1, GPDH; MgsA, GloA-B, AldA-B, GRE2; GPDH, PLG, RisA-B, TK, TA, FPK, XPK, PglD, OpcA; AlaDH, AlaR, AlaGAT, SerD, SerT; *glcB*-*F*, HxK, GoxR, HpyrR, GalDH, AldDH, 2PGP; PyK, PpS, PpD, Pc, PEPC, ME; PDE, POX, PDC, PFORa-d, PDHA-B, DDH, OPOT, ADHA; GOX, *lysR*
Lipid/pigments metabolism	Chl, iron tetrapyrrols, fatty acids, isoprenoids, phospholipids	PMgCD, PMgCH, PmgMT, ChlEAe, DVR, ChlB, ChlG, ChlL-M, POR; GltR, UroM, UroD, HemQ, HemX-Y; FabA-T, HpnE-H; CruA-G, CrtL, GlyP, GarL
Carbon substrate intermediary metabolism	Calvin cycle; fructose, galactose, mannose, sucrose, polyglucoside, aminosugar, nucleotide sugar, C_1_-substrate and glycogen metabolism	PRK, Rbc, PGK, GAPDH, TPI, FBA1-2, FBP_I-X, TK, RPE; *cbbL*; *cbbS*, Ss, RBCS, RBCI, ClCP, CA; ManA-P, MalE-G, K; MsmK, LamB, MalL-K, MalA-B; NAGK1-3, NagA-E, CbSA, ChbA-C; mtdA, FTCLI; GAT_C, GAT_D, GS, GBr, GP, MP, MOTs, aAMP
Nitrogen substrate intermediary metabolism	Nitrogen and ammonia assimilation; urea cycle	*cynT, cynR, cynS, cynX*; *nrfB-H*, *niR1-3, niTa-Tc*, *narC, narG, narH, narI*, *napA-L, napR-T*, *nrfE-G, nrfX*, GsI, GSIII, GlnE, GlnD, GOGDP1, GOGDP2, GlxC-D, GOGD, GAT, NRI, NRII, PII, PIIK, NtcA; UreD-G
Protein metabolism	Amino acids, polyamines and glutathione biosyntheses; protein processing, degradation, modification and folding; selenoproteins	GltB, GlxC-B, GldH, AspA-C, AsnA-B, GltS, GlsA, HisA-I, AstA-E, ArgR, SpeA-C, ArcA-D, MetN-T, ThrA-C, AspC, CysB-E, Lys1, LLP, CadA-C, DavA-D, CodA, LeuA-D, TrpA-E, TyrA, PheA, ProA-C, SelD, GlyA-B, AlaB, AlaR, CsdA, SufS, SerA-C; SelA-B
Mineral substrate metabolism	Phosphate, sulfur, iron and potassium metabolism	*pho* regulon; high-affinity phosphate transporter genes; siderophores; bacterioferritins; CysA, CysQ, SAT1-2, APSR, ASK, SIRFP, FPR_A; FhuB; *kdpA-E*, KefA-B, KefF
Enzyme cofactor metabolism	Coenzyme B_12_, FAD, FMN, lipoic acid, Mo-cofactor, NAD, pterines, pyridoxin, quinone, riboflavin, thiamine biosynthesis	BioC, BioH, HoxE, HoxF, HoxH, HoxU, HoxY, CobA-C, CbiA-K, ThiB-G; UbiA-H; *menA-D*; PyrD, PyrR, PyrP, RSAe, FMNAT, LUMP, RK, RSA, *gapA*, *pdxA-K, *FolA-B; LipA-C, LipL-M, BirA, GlyP, PdhB, SucB, AceB, BkdB
Secondary metabolism	Auxin, flavonoids, terpenes and derivatives biosynthesis	plant hormones (AUX1, APRT, PRAI, IGS,TSa, TM, IAH, IAD, AAD, AFTS), toxin-antitoxin replicon stabilization systems (RelB, E, F; CcdA-B, ParE-D, HigA-B, VapC-B, YoeB, YefM, YafQ, DinJ, YeeU, YkfI, YafW, YpjF, YgiZ)
Membrane transport		ABC transporters (*phnC-E*, *oppA-F*, *dppA-F*), FtsY, TatA-E, MgtA-E, YcnL-K, CopC-D, CsoR, CopA, ModB; TolA, TonB, NikQ, NikM, CbiQ, CbiO, CbiM, BioM, BioN, MtsA-C, YkoC-E lipT, Sec-translocase; secretion protein type E, type IV pilus (pilA, pilT)
Cell division, cytoskeleton		*ftsZ*, *ftsW, ftsB*, *ftsL, ftsA,* ZipA, ZapA, MinC-E, ParA-B, Maf, YgiD, YeaZ(TsaB); MreB-D, RodA, MraZ
Regulation		*kaiA-C*, *sasA*, CikA, Pex, CPM; *nrrA*, *groEL*, *grpE, dnaJ,* LdpA, PSF, SigB, RsbR-W, PemK, SigF, SigG, SigFV, sig70, *hetR,* TyrR*,* IcsR, YbeD*,* cAMPB, FNR, CGA, *dnaG, rpoD*, *exoY*, *pagA*, AtxA, AtxR*, hcnA-C,* Clp2, ArsR, HisI, PyrC, FolE, HemB, CynT, CysS, YGR262c; SpoT, RelA, Rex, Fur_Zur, Fnr, gpp
Stress response	Protection from reactive oxygen species; oxidative and periplasmic stress	*sodA-C*, cyt c551 peroxidase, HP1; SoxS-R, OxyR, PerR, NnrS, AhpC, HemO, *gshA-B*, GltC, GltT, Rth, SOR, Rdx, ROO, NRO, AHR, grlA, EnvC, HbO, CHb, FHP, HmpX, Hfq, HflX-C; DegP-S, RseP, RseA-B, SurA, DegQ, HtrA
Phages, integrons and CRISPRs		SA bacteriophages 11, TFP1-2, TFAP, TFC, Lys1-8, LysA-B, Hol1-2, TransI, endolysin; integrons (Int1-2, Int4, InyIPac); CRISPR cmr-cluster (Cmr1-6, Csx11, NEO113, TM1812, Cas02710); CasReg, Cas1-7, Csh1-2, Csd1-2, Cse1-4, Csn1-2, Csy1-4, Csa1-5, Csm1-5, Cst1-2

Chl *a*/*b*-containing *Prochlorothrix* and *Prochloron* were long considered to have a common ancestry with chloroplasts of green algae and higher plants [27, 28]. However, *P. hollandica* and another prochlorophytes were shown to possess unique genes *pcbA* − *pcbC* coding chl *a*/*b*-LHC apoproteins and they are dissimilar from CAB apoprotein superfamily of chloroplast antenna [19–30]. It is notable that we found some PS II proteins commonly absent in cyanobacteria but usually belonging to chloroplast in green algae and higher plants: PsbW (6.1 kDa, nuclear encoded), PsbT (5 kDa, nuclear encoded), PsbR (10 kDa) and PsbQ (16 kDa, oxygen evolving complex protein). We also found that *P. hollandica* contains an ortholog of *hetR* gene (key regulator of heterocyst differentiation) although all these filamentous non-heterocystous cyanobacteria are devoid of nitrogenase and other prerequisites for diazotrophy [31, 32].

## Conclusions

The studying of *P. hollandica* CCAP1490/1^T^ (CALU1207) genome is valuable for analyses of photosynthesis genes evolution and for comparative genomics of cyanobacterial adaptation.

## Abbreviations

chl: Chlorophyll
HL: High light
LHC: Light-harvesting complex
LL: Low light
PBP: Phycobiliprotein
PS: Photosystem
